# Clinical outcomes of quadriceps, hamstring, and bone–patellar tendon–bone autografts for ACL reconstruction: a meta-analysis of randomized controlled trials

**DOI:** 10.1186/s43019-026-00320-w

**Published:** 2026-05-07

**Authors:** Riccardo D’Ambrosi, Luca Farinelli, Piero Franco, Andrea Marchetti, Amit Meena, Danko Dan Milinkovic, Srinivas BS Kambhampati, Luca Maria Sconfienza, Thomas Patt, Chiara Ursino, Elisabeth Abermann, Christian Fink

**Affiliations:** 1 IRCCS Ospedale Galeazzi - Sant’Ambrogio, Milan, Italy; 2 Dipartimento di Scienze Biomediche per la Salute, Università degli Studi di Milano, Milan, Italy; 3 Marche Polytechnic University, Ancona, Italy; 4 IRCCS INRCA, Ancona, Italy; 5 Azienda Ospedaliero-Universitaria Careggi, Florence, Italy; 6 University of Trieste, Trieste, Italy; 7 Shalby Hospital, Jaipur, India; 8 Charité - University Medicine Berlin, Berlin, Germany; 9 Sri Dhaatri Orthopaedic, Maternity & Gynaecology Center SKDGOC, Vijayawada, India; 10 Bergman Clinics, The Hague, Netherlands; 11 Gelenkpunkt, Innsbruck, Austria; 12 Research Unit for Orthopaedic Sports Medicine and Injury Prevention (OSMI), Private University for Health Sciences Medical Informatics and Technology, Innsbruck, Austria

**Keywords:** Anterior cruciate ligament reconstruction, Quadriceps tendon autograft, Hamstring tendon autograft, Bone–patellar tendon–bone autograft, Donor-site morbidity, Graft failure, Randomized controlled trials, Meta-analysis

## Abstract

**Background:**

The quadriceps tendon (QT) has emerged as a reliable autograft for anterior cruciate ligament reconstruction (ACLR), but uncertainty remains regarding several key comparative aspects—particularly donor-site morbidity, long-term graft survival, knee stability, and complication rates—when evaluated against hamstring tendon (HT) and bone–patellar tendon–bone (BPTB) autografts. High-level evidence restricted to randomized controlled trials directly comparing QT with HT or BPTB remains limited. To compare clinical outcomes, graft failure, donor-site morbidity, and knee stability among QT, HT, and BPTB autografts for primary ACLR using level-I and level-II randomized controlled trials (RCTs).

**Methods:**

The MEDLINE (PubMed), Embase (Elsevier), and Cochrane Library databases were searched on 1 September 2025, and repeated 2 weeks later. Only level-I or -II RCTs comparing QT to HT or BPTB in primary ACLR were included. Random-effects meta-analyses were performed for International Knee Documentation Committee (IKDC) and Lysholm scores, instrumented laxity, graft failure, donor-site morbidity, and reoperation. Risk of bias was assessed with RoB 2.0, and small-study effects with funnel and doi plots.

**Results:**

Eleven RCTs (mean follow-up, 2–10 years) were included. Pooled IKDC scores averaged 84.8 (95% CI 81.9–87.9) and Lysholm scores averaged 93.1 (95% CI 91.6–94.6), with no significant differences between QT and either comparator (*P* > 0.05). Side-to-side anterior tibial translation averaged 1.2 mm (95% CI 0.99–1.54 mm) across all grafts, also without significant differences (*P* > 0.05). Pooled graft failure and ipsilateral reoperation rates were 0.7% (95% CI 0.0–1.9%) and 2.3% (95% CI 0.6–4.7%), respectively, again with no between-graft differences (*P* > 0.05). Donor-site morbidity did not differ significantly between QT and HT (mean 13.83 [95% CI 9.6–19.83]; *P* > 0.05).

**Conclusion:**

This meta-analysis of level-I/II randomized controlled trials found no statistically significant differences among quadriceps tendon, hamstring tendon, and bone–patellar tendon–bone autografts in patient-reported outcomes, knee stability, graft re-rupture, or additional knee surgery. Donor-site morbidity comparisons were limited by incomplete reporting, particularly for BPTB. These findings suggest that contemporary surgical techniques and rehabilitation protocols may minimize graft-specific differences in mid-term outcomes, although interpretation should consider the limited number of direct comparative trials across all three graft types.

*Level of evidence* Systematic review and meta-analysis; level of evidence, 1 and 2.

**Supplementary Information:**

The online version contains supplementary material available at 10.1186/s43019-026-00320-w.

## Introduction

Anterior cruciate ligament (ACL) injuries are prevalent among physically active and young people with a high incidence in sports-related activities. The ACL plays a critical role in maintaining knee stability. Its rupture can lead to significant functional impairment, chronic instability, and an increased risk of early-onset osteoarthritis [1].

Surgical reconstruction remains the gold standard treatment for symptomatic ACL tears, especially in young and athletic populations. Autografts are widely used in ACL reconstruction (ACLR), with the most common options being bone–patellar tendon–bone (BPTB), hamstring tendons (HT), and, more recently, the quadriceps tendon (QT), with or without a bone block [2].

The QT has gained increasing attention owing to its favourable biomechanical properties, including a larger cross-sectional area of collagen structure compared to patellar tendon, making it suitable for both primary and revision ACLR—also in elite athletes [3–8].

In addition to the growing interest in QT, both HT and BPTB autografts remain widely used and well-established options for ACL reconstruction. HT autografts are favored for their low donor-site morbidity, reduced anterior knee pain, and smaller incision requirements; however, concerns have been raised regarding variable graft diameter, slower tendon-to-bone healing, and potential postoperative hamstring weakness [9–14]. Conversely, BPTB autografts provide the advantage of rapid bone-to-bone healing and excellent long-term stability but are consistently associated with a higher risk of anterior knee pain, kneeling discomfort, and extensor mechanism complications [9–14]. These well-documented trade-offs highlight the need for high-level comparative evidence to determine whether any clinically meaningful differences exist among QT, HT, and BPTB autografts.

This increasing clinical adoption of the QT autograft has been recognized by experts in the field and contributed to the establishment of the International Quadriceps Tendon Interest Group (IQTI) in 2017 [5, 8, 15–17].

Comparative studies have shown that QT autografts are associated with lower donor-site morbidity than BPTB, while providing comparable outcomes in terms of knee stability and return to sport [10, 14].

Recent refinements in graft harvesting and tunnel preparation techniques have aimed to improve safety and reproducibility in ACL reconstruction, including for QT autografts. However, variations in surgical technique across studies—such as harvest approach or tunnel configuration—may influence postoperative outcomes and represent potential sources of heterogeneity in the existing literature [18–21].

Despite several previous systematic reviews comparing QT to HT and BPTB grafts, consensus remains limited because many of these analyses included predominantly retrospective studies, combined heterogeneous graft types and surgical techniques, and incorporated highly variable rehabilitation protocols. Moreover, only a small number of high-quality randomized controlled trials were available, reducing the strength of prior comparative conclusions [22, 23].

Therefore, the aim of the present systematic review and meta-analysis was to analyse clinical outcomes, laxity, donor-site morbidity and revision rate of QT compared to BPTB and HT graft in ACLR in randomized controlled trials.

## Material and methods

A systematic search strategy was developed according to the Preferred Reporting Items for Systematic Reviews and Meta-Analyses (PRISMA) guidelines and is registered in the PROSPERO Registry—CRD420251125882 [24, 25]. The methodological quality of the review was assessed using the AMSTAR-2 checklist [26]. An electronic database search was performed to identify randomized controlled trials (RCTs) comparing QT, HT, and BPTB autografts for anterior cruciate ligament reconstruction, reporting clinical outcomes such as International Knee Documentation Committee (IKDC), Lysholm score, anterior tibial translation, donor-site morbidity, graft re-rupture, and new knee surgeries.

The MEDLINE (PubMed), Embase (Elsevier), and Cochrane Library databases were searched on 1 September 2025, and repeated 2 weeks later.

A comprehensive search of MEDLINE (PubMed), Embase, and the Cochrane Library was conducted using a structured combination of MeSH terms and free-text keywords. In PubMed, MeSH terms included “Anterior Cruciate Ligament”, “ACLR”, “Quadriceps Tendon”, “hamstring”, and “Patellar Tendon”. These MeSH terms were combined with free-text keywords commonly used in ACL reconstruction (ACLR) literature, including “hamstring”, “hamstring tendon”, “semitendinosus”, “gracilis”, “quadriceps tendon autograft”, and “bone–patellar tendon–bone (BPTB)”, as well as study-design terms such as “randomized controlled trial”, “randomised controlled trial”, and “controlled clinical trial”. Embase and Cochrane searches applied database-specific subject headings along with the same ACLR-, graft-, and RCT-related keywords. To ensure methodological clarity and avoid ambiguity across databases, MeSH terms and keywords were applied and combined separately in each database.

### Eligibility criteria

The literature selected for this study was based on the following criteria:

### Study design

Only randomized clinical trials (RCTs) were included in the systematic review and meta-analysis.

### Participants and interventions

Randomized clinical trials conducted in skeletally mature patients who underwent ACL reconstruction using QT compared to HT or BPTB grafts. Skeletally mature patients were defined a priori as individuals with closed physes. Accordingly, we included only studies enrolling subjects described as skeletally mature or managed with standard ACL reconstruction techniques, without the use of physeal-sparing or physeal-respecting procedures designed for patients with open growth plates.

### Type of outcome measures

Six primary outcomes were extracted:

- *The IKDC subjective knee form*: A validated patient-reported outcome measure assessing symptoms, knee function, and sports activity, with scores ranging from 0 to 100; higher scores indicate better function [27]
- *Lysholm score*: A patient-reported outcome measure evaluating symptoms and functional limitations after knee ligament injury. It comprises eight items—pain, instability, locking, swelling, stair climbing, squatting, limp, and need for support—yielding a total score from 0 to 100, with higher scores reflecting better knee function [28]
- *Anterior tibial translation*: Measured as the side-to-side difference in anterior tibial translation (mm) using the KT-1000 arthrometer [29]
- *Donor site morbidity*: Assessed with the “donor site–related functional problems following ACL reconstruction” score, which includes 16 items weighted from 0 (no morbidity) to 6. The total score is aggregated and normalized to 0–100, where 0 represents no donor-site issues and 100 indicates the worst possible outcome across all questions [11]
- *ACL re-rupture:* Re-injury of the ipsilateral anterior cruciate ligament.
- *New knee surgeries:* Any additional surgical procedures performed on the ipsilateral knee previously operated on for ACL reconstruction.

## Data collection and analysis

### Study selection

The retrieved articles were first screened by title and, if found relevant, further screened by reading the abstract. After excluding studies that did not meet the eligibility criteria, the entire content of the remaining articles was assessed for eligibility. To minimize the risk of bias, the authors reviewed and discussed all the selected articles, references, and articles excluded from the study. In case of disagreement among the reviewers, the senior investigator made the final decision. At the end of the process, additional studies that might have been missed were searched manually by going through the reference lists of the included studies and relevant systematic reviews.

### Data collection process

Data were extracted from the selected articles by the first two authors using a computerized tool created with Microsoft Access (Version 2010, Microsoft Corp., Redmond Washington). Each article was validated again by the first author before analysis.

### Level of evidence

The Oxford Levels of Evidence set by the Oxford Centre for Evidence-Based Medicine were used to categorize the level of evidence [30].

### Risk of bias assessment

The methodological quality of the included randomized controlled trials was assessed using the Cochrane Risk of Bias 2.0 (RoB 2) tool [31].

This tool evaluates the internal validity of each trial across five domains:

- Bias arising from the randomization process,
- Bias due to deviations from the intended interventions,
- Bias due to missing outcome data,
- Bias in measurement of the outcome, and
- Bias in selection of the reported result.

Each domain was rated as “low risk of bias”, “some concerns”, or “high risk of bias”, and an overall risk-of-bias judgement was then derived according to Cochrane guidance. Two reviewers performed the assessment independently, and any disagreements were resolved by consensus.

### Statistical analysis

For IKDC, Lysholm, KT-1000, Donor Site Morbidity Score, we performed a meta-analysis with a random-effect model on log transformed means using the restricted maximum-likelihood (REML) estimator for variance estimation. The pooled estimates were presented as pooled means with 95% confidence intervals (CIs). The meta-analysis included primary studies with available standard deviation (SD) or range, from which we estimated the SD [32].

Continuous outcomes (IKDC, Lysholm, KT-1000) were analyzed on the log scale because several studies demonstrated non-normal distributions, right-skewed score ranges, and heterogeneity in variances. Log-transformation is commonly used to stabilize variances and reduce skewness, providing estimates more consistent with the assumptions of parametric meta-analytic models. Back-transformed results were presented for clinical interpretability.

A meta-analysis on the frequency of re-rupture and new knee surgeries were conducted using a random-effects model with the DerSimonian-Laird estimator for the variance. The raw proportions were stabilized using the Freeman–Tukey double arcsine transformation. The pooled estimates were presented as pooled proportions with corresponding 95% CIs.

Differences among groups were explored with mixed-effects meta-regression models with common between-study variance in subgroups, using variance estimators and transformations previously described. For each outcome, we tested within and between group heterogeneity with a Cochran’s *Q* test. Group comparisons were performed with the group “Quadriceps” as reference category.

Between-study variations were assessed for each model with the Cochran’s *Q* test of heterogeneity and the Higgins *I*^2^ statistic. Statistical heterogeneity was defined as substantial if *I*^2^ > 50% [33]. Publication bias and small-study effect were assessed through a funnel plot and doi plot. Funnel plot symmetry was tested with rank correlation test and the regression test while the LKF index was calculated with the doi plot. A sensitivity analysis with Trim-and-fill method was performed and the fail-safe *N* was calculated using the Rosenthal approach. Detailed analysis are reported in Supplementary Material.

Two-tailed tests were performed. A *P*-value of < 0.05 was considered to indicate statistical significance. The analysis was carried out using R (version 4.3.0, R Foundation for Statistical Computing, Vienna, Austria. URL https://www.R-project.org/) specifically with meta (version 8.0.1) and metafor packages (version 4.2.0).

## Results

**Fig. 1:**
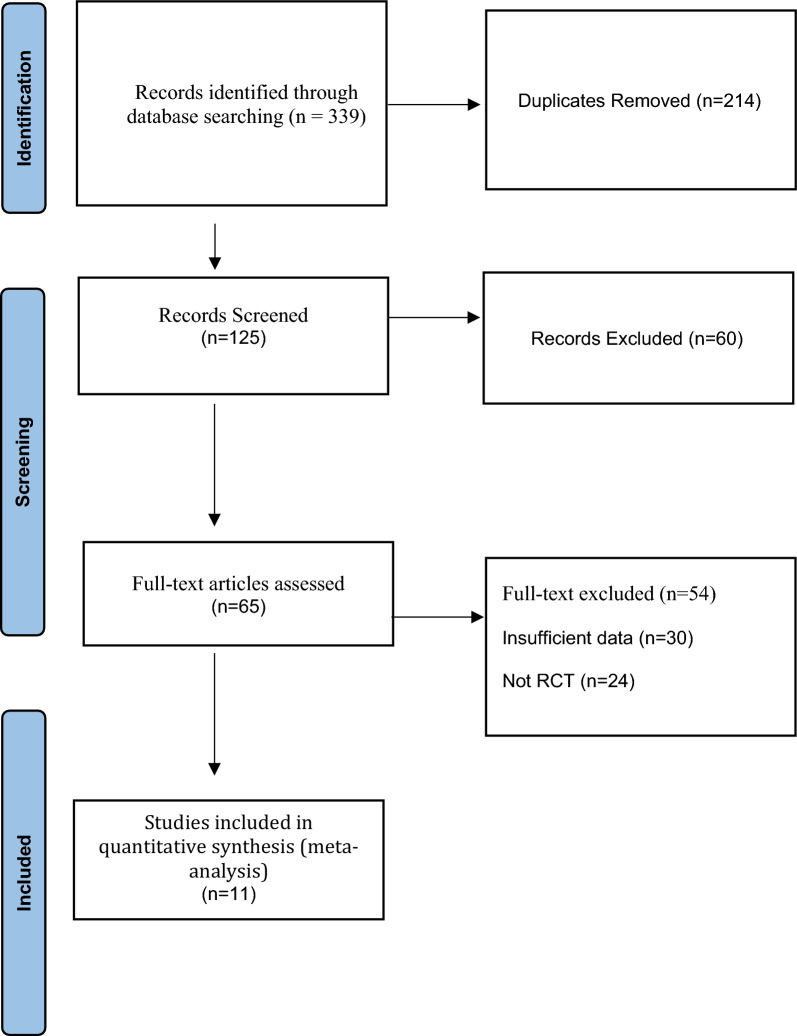
Preferred Reporting Items for Systematic Reviews and Meta-analyses (PRISMA) flow chart indicating the inclusion of research articles for final analysis

A total of 339 records were identified through the initial database search. After removal of 214 duplicates, 125 titles and abstracts were screened. Of these, 60 records were excluded, leaving 65 full-text articles for eligibility assessment. Following full-text review, 54 studies were excluded, and 11 articles met the inclusion criteria and were retained for the final analysis [13, 34–43]. The study selection process is summarized in the PRISMA flow diagram (Fig. 1).

### Risk of bias assessment

According to the RoB 2.0 tool, most included randomized controlled trials showed low risk of bias in the domains of randomization process and deviations from intended interventions. Some studies demonstrated some concerns regarding measurement of outcomes and selection of the reported results, primarily due to incomplete reporting of assessor blinding or protocol registration. A detailed domain-level assessment for each study is provided in the Supplementary Material.

Details of included studies are reported in Table 1.

**Table 1.** Summary of included randomized controlled trials comparing quadriceps, hamstring, and patellar tendon autografts for ACL reconstruction

Author	Number of patients	Gender (M/F)	Age	Follow-up	IKDC	Lysholm	KT-1000 (mm)	Re-rupture	Donor site morbidity	New knee surgeries (excluding ACL revision)
Quadriceps										
Bariè et al. 2020 [34]	21	n.a	30.5 ± 7.8 (15–49)	10 years	92 ± 11.5 (60–100)	95.6 ± 7.8 (74–100)	1.00 ± 1.095	0	n.a	1
Vilchez-Cavazos et al. 2020 [43]	14	n.a	23 (19.5–30.5)	1 year	90 (83–91.5)	95 (91–100)	n.a	0	n.a	0
Ebert et al. 2024 [36]	48	n.a	28.1 ± 8.2 (16–47)	2 years	89.1 ± 9.6	92.5 ± 8.0	1.2 ± 1.1	1	9.3 ± 10.6	6
Horstmann et al. 2022 [13]	21	n.a	24.1 ± 3.6	2 years	89.3 ± 12.2	90.4 ± 11.9	0.7 ± 1.1	3	n.a	1
Calvert et al. 2024 [35]	48	n.a	28.1 ± 8.2	2 years	89 ± 10	93 ± 8	n.a	0	n.a	0
Lind et al. 2020 [38]	44	n.a	27.2 ± 6.4	2 years	82 ± 14	n.a	1.8 ± 1.0 (measured at 1 year)	1	14 ± 17	4
Lund et al. 2014 [39]	21	n.a	30 ± 9	2 years	84 ± 13	n.a	1.1 ± 1.4	0	n.a	1
Martin-Alguacil et al. 2018 [40]	19	n.a	18.7 ± 3.6	2 years	n.a	n.a	n.a	1	n.a	0
Komzák et al. 2022 [37]	40	21/19	29.8	28 months	78.3 ± 10.5 (65.5–93.1)	90.1 ± 6.7 (76–100)	n.a	0	n.a	0
Sinding et al. 2020 [41]	42	25/17	28.7 ± 6.4	1 year	76 ± 17	n.a	n.a	0	n.a	0
Tang et al. 2024 [42]	17	n.a	28.06 ± 6.24	2 years	87.12 ± 6.61	90.00 ± 4.26	n.a	0	n.a	0
Hamstring										
Vilchez-Cavazos et al. 2020 [43]	14	n.a	23 (20–30)	1 year	90 (87–93)	98 (90–100)	n.a	0	n.a	0
Ebert et al. 2024 [36]	49	n.a	29.4 ± 7.7	2 years	91.7 ± 7.5	94.4 ± 6.6	1.1 ± 0.8	0	12.3 ± 10.7	5
Horstmann et al. 2022 [13]	26	n.a	32.7 ± 11.4	2 years	83.7 ± 12.7	83.5 ± 17.4	0.2 ± 2.2	2	n.a	0
Calvert et al. 2024 [35]	49	n.a	29.4 ± 7.7	2 years	92 ± 8	94 ± 7	n.a	0	n.a	0
Lind et al. 2020 [38]	48	n.a	27.1 ± 6.1	2 years	78 ± 18	n.a	1.9 ± 1.7 (measured at 1 year)	1	22 ± 18	5
Martin-Alguacil et al. 2018 [40]	17	n.a	19.2 ± 3.6	2 years	n.a	n.a	n.a	3	n.a	0
Sinding et al. 2020 [41]	43	23/20	28.3 ± 6.2	1 year	76 ± 15	n.a	n.a	0	n.a	0
Tang et al. 2023 [42]	16	n.a	28.31 ± 8.55	2 years	90.13 ± 6.99	90.06 ± 7.33	n.a	0	n.a	1
Patellar tendon										
Bariè et al. 2020 [34]	22	n.a	30.6 ± 7.5 (18–48)	10 years	91 ± 7.3 (75–100)	95.2 ± 6.6 (75–100)	1.05 ± 1.36	0	n.a	3
Lund et al. 2014 [39]	18	n.a	30 ± 9	2 years	70 ± 16	n.a	0.8 ± 1.7	1	n.a	2
Komzák et al. 2022 [37]	40	23/17	31.0	28 months	74.2 ± 10.2 (55–80)	93.4 ± 9.6 (64–100)	n.a	0	n.a	0

The table reports the main demographic, surgical, and clinical outcome data extracted from each included study.

Variables are reported as mean ± standard deviation (range) or as otherwise specified.

*IKDC*  International Knee Documentation Committee subjective score, *Lysholm*  Lysholm Knee Scoring Scale, *KT-1000*  side-to-side anterior laxity (mm) measured with KT-1000 arthrometer, *n.a*, not applicable,*VAS*  Visual Analog Scale.

Level of evidence of the articles evaluated using Rob 2 are reported in Table S1.

### The International knee documentation committee (IKDC) subjective knee form

**Fig. 2:**
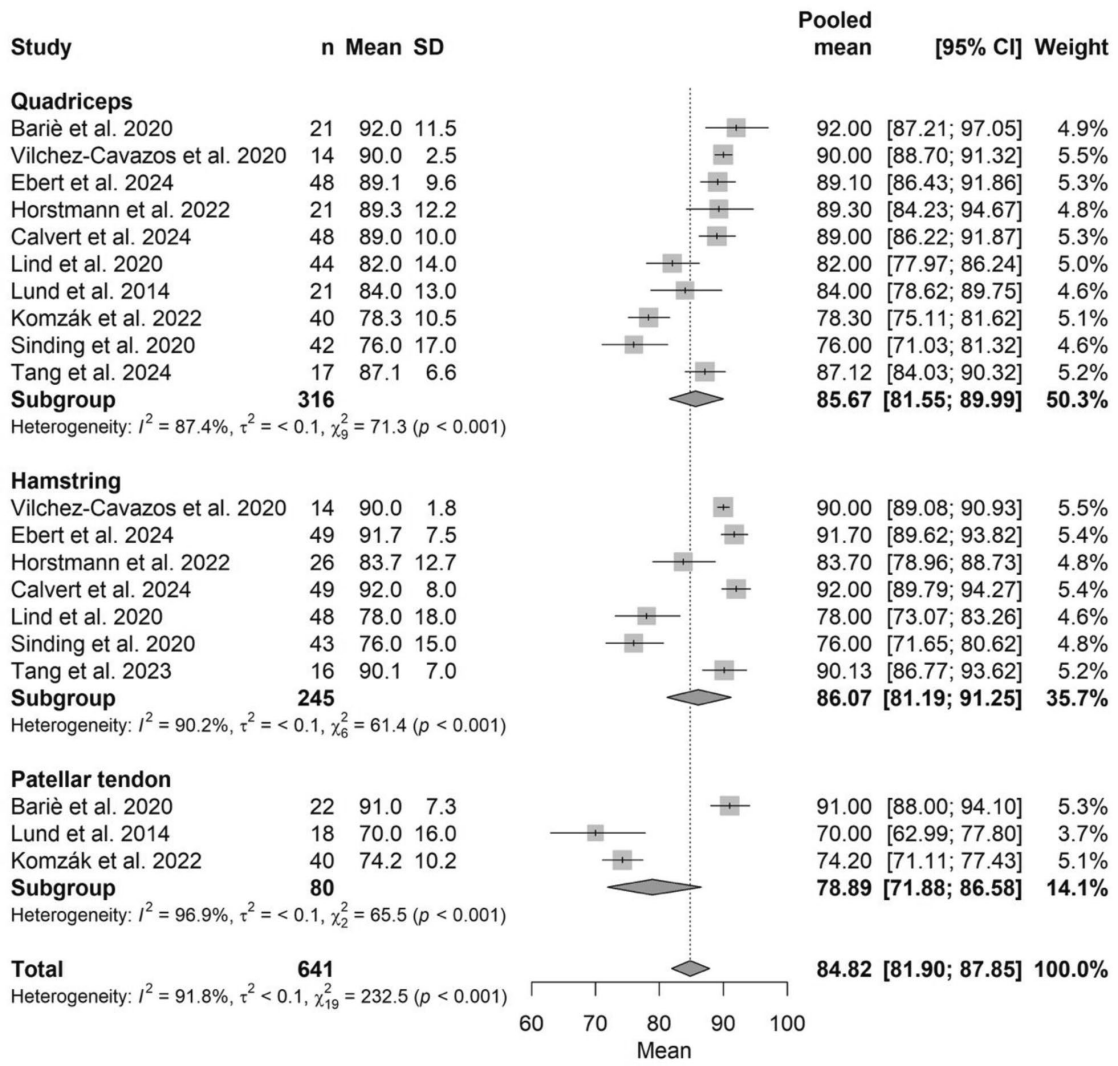
Forest plot of pooled IKDC scores comparing ACL autograft types

The pooled IKDC score was 84.82 (95% CI 81.90–87.85), with no significant differences among graft types (*P* > 0.05). Scores ranged from 78.89 (95% CI 71.88–86.58) for BPTB to 86.07 (95% CI 81.19–91.25) for HT. The corresponding forest plot is presented in Fig. 2.

### Lysholm score

**Fig. 3:**
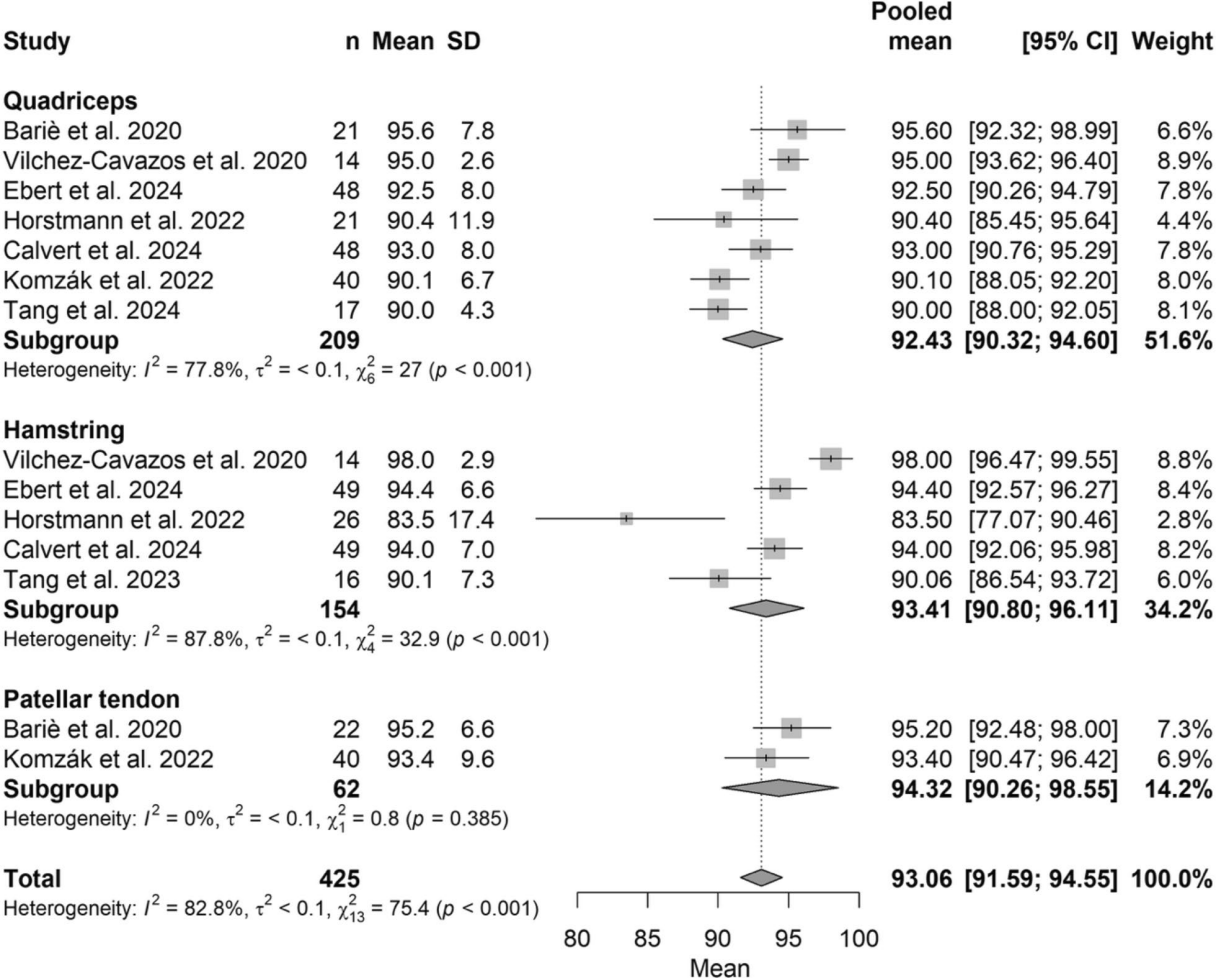
Forest plot of pooled Lysholm scores comparing ACL autograft types

The pooled Lysholm score was 93.06 (95% CI, 91.59–94.55), with no significant differences among graft types (*P* > 0.05), ranging from 92.43 (95% CI, 90.32–94.60) for QT to 94.32 (95% CI, 90.26–98.55) for BPTB. The corresponding forest plot is presented in Fig. 3.

### Anterior tibial translation

**Fig. 4:**
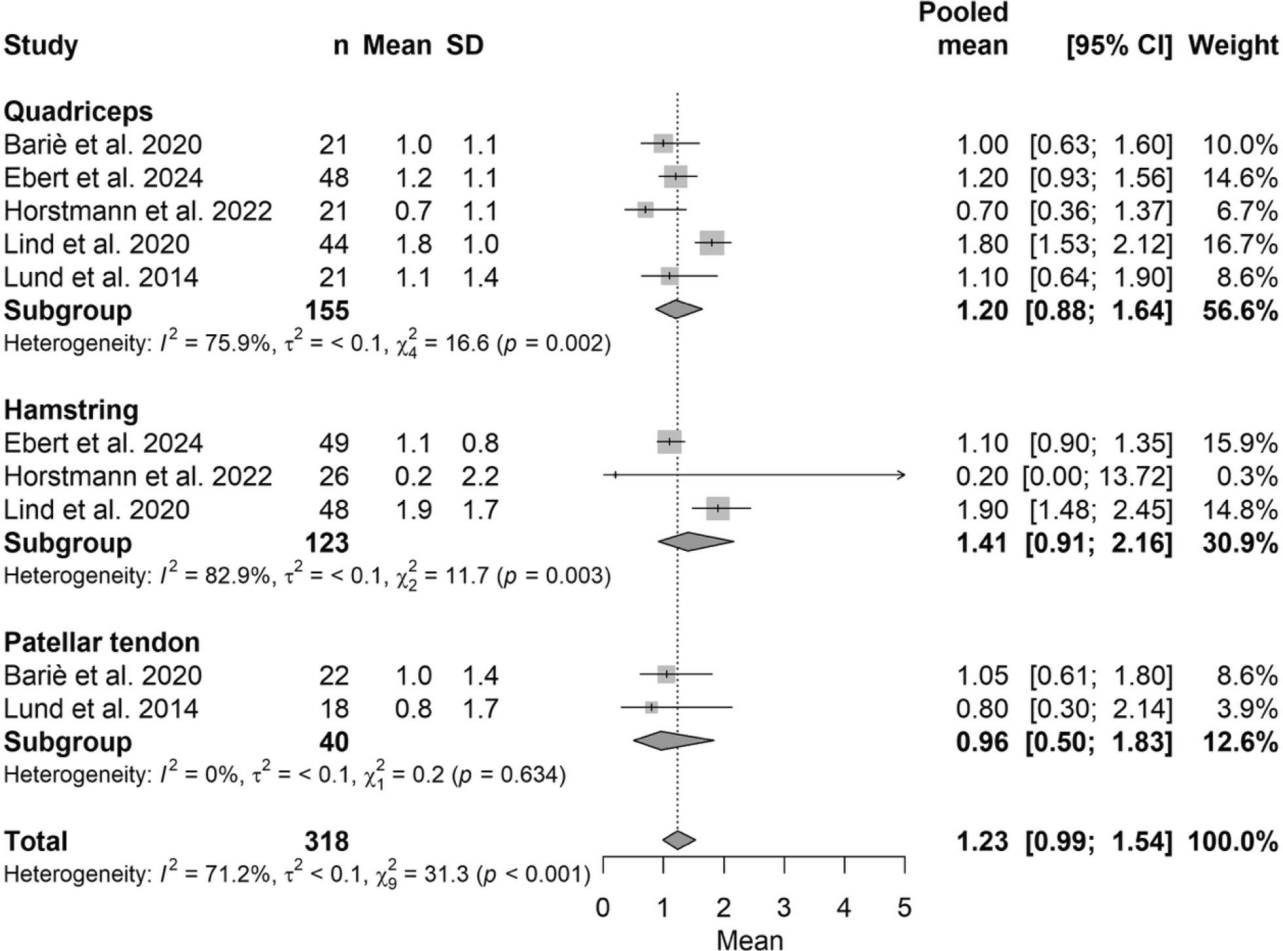
Forest plot of anterior tibial translation measured with KT-1000 comparing ACL autograft types

The pooled anterior tibial translation, measured with the KT-1000 arthrometer in millimeters, was 1.23 (95% CI 0.99–1.54), with no significant differences among graft types (*P* > 0.05), ranging from 0.96 (95% CI 0.50–1.83) for BPTB to 1.41 (95% CI 0.91–2.16) for HT. The corresponding forest plot is shown in Fig. 4.

### Donor site morbidity

**Fig. 5:**
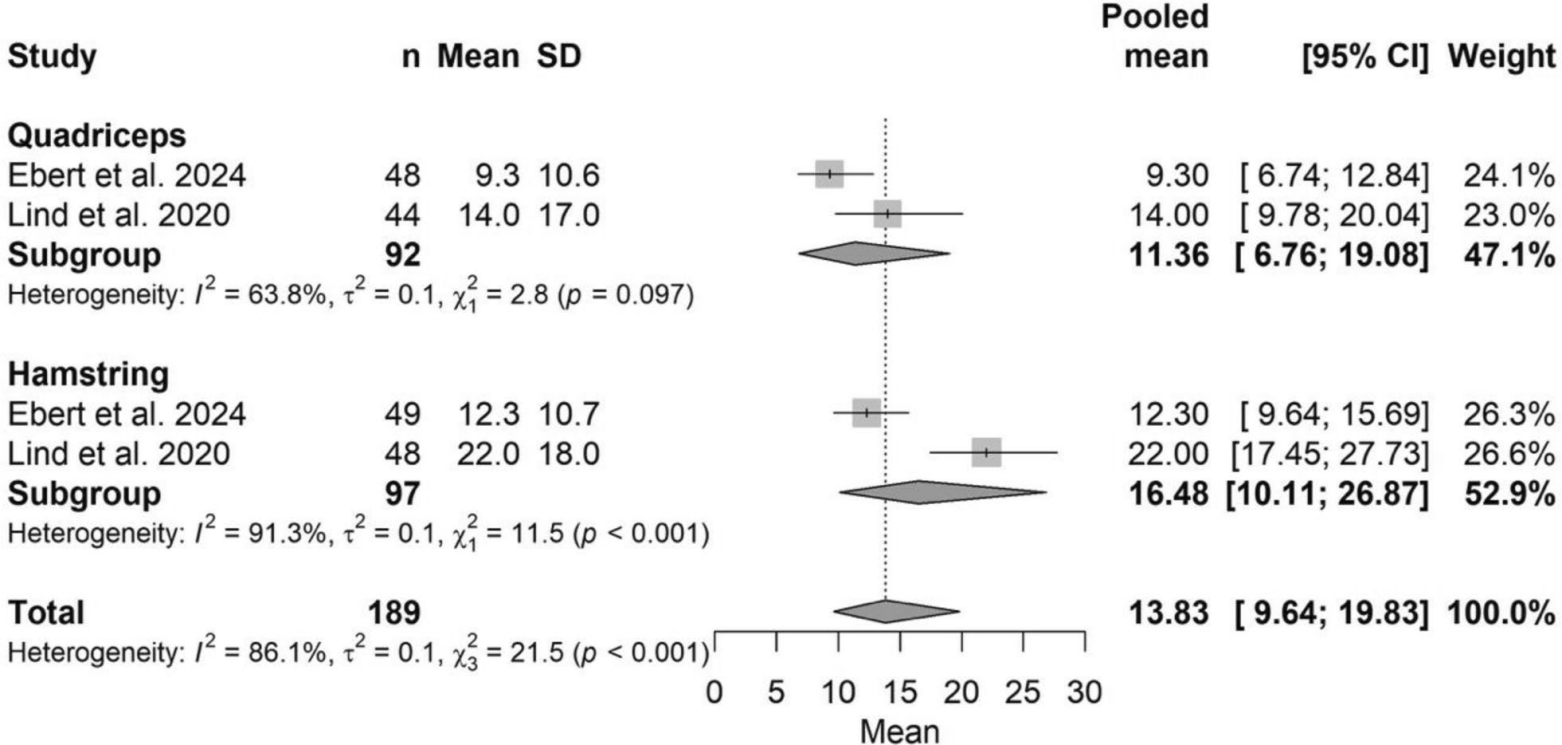
Forest plot of donor-site morbidity comparing quadriceps and hamstring tendon autografts

The pooled donor-site morbidity was 13.83 (95% CI 9.64–19.83), with no significant difference between quadriceps and HT grafts (*P* > 0.05). The corresponding forest plot is shown in Fig. 5.

### ACL re-rupture

**Fig. 6:**
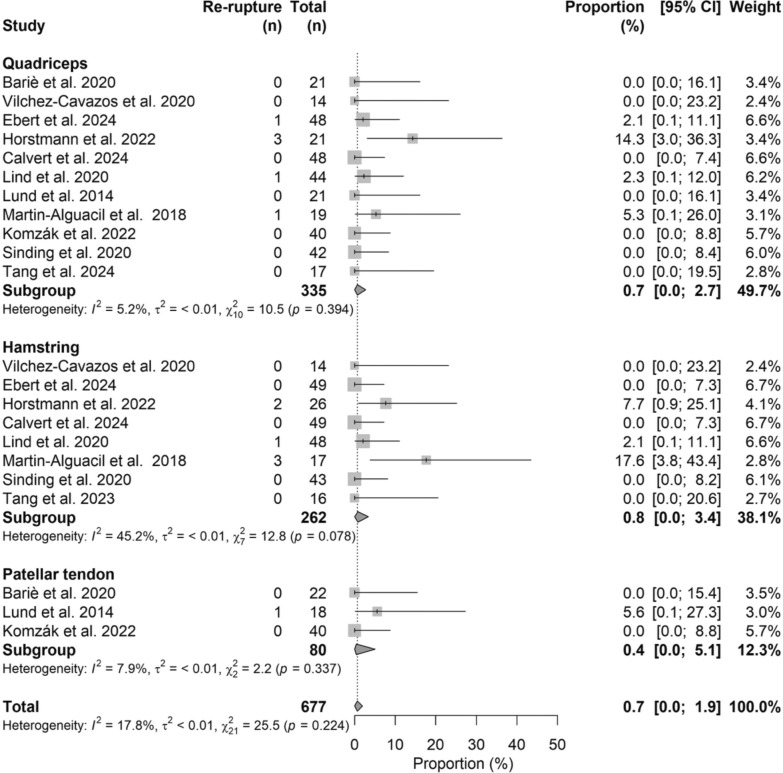
Forest plot of re-rupture rate comparing ACL autograft types

The pooled re-rupture rate was 0.7% (95% CI 0.0–1.9%), with no significant differences among graft types (*P* > 0.05), ranging from 0.4% (95% CI 0.0–5.1%) for BPTB to 0.8% (95% CI 0.0–3.4%) for HT. The corresponding forest plot is shown in Fig. 6.

### Odds ratio

**Fig. 7:**
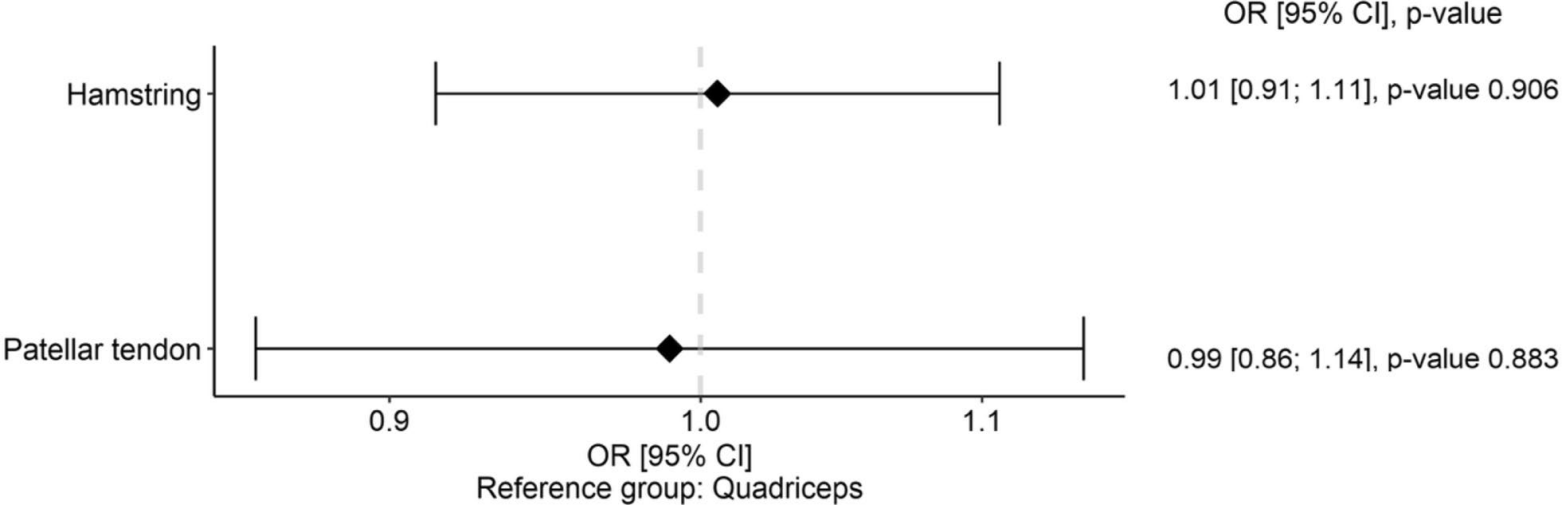
Forest plot of odds ratios (OR) for ACL re-rupture

The pooled odds ratio (OR) analysis revealed no significant differences (*P* > 0.05) among graft types for the risk of re-rupture, ranging from OR = 0.99 (95% CI 0.86–1.14) for BPTB to OR = 1.01 (95% CI 0.91–1.11) for HT. The corresponding forest plot is presented in Fig. 7.

### New knee surgeries

**Fig. 8:**
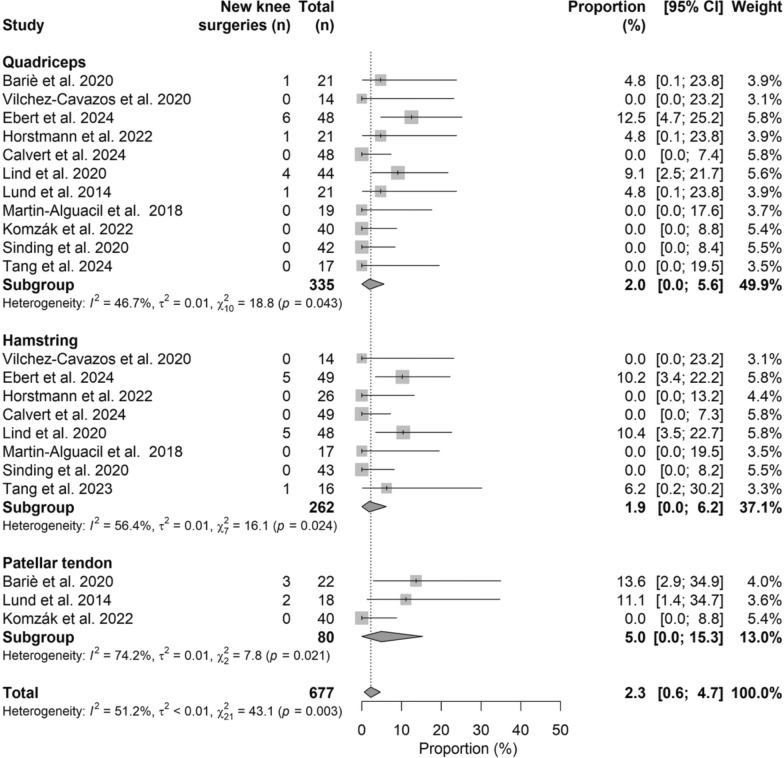
Forest plot of new knee surgeries rate comparing ACL autograft types

The pooled rate of new knee surgeries was 2.3% (95% CI 0.6–4.7%), with no significant differences among graft types (*P* > 0.05), ranging from 1.9% (95% CI 0.0–6.2%) for HT to 5.0% (95% CI 0.0–15.3%) for BPTB. The corresponding forest plot is shown in Fig. 8.

### Odds ratio

**Fig. 9:**
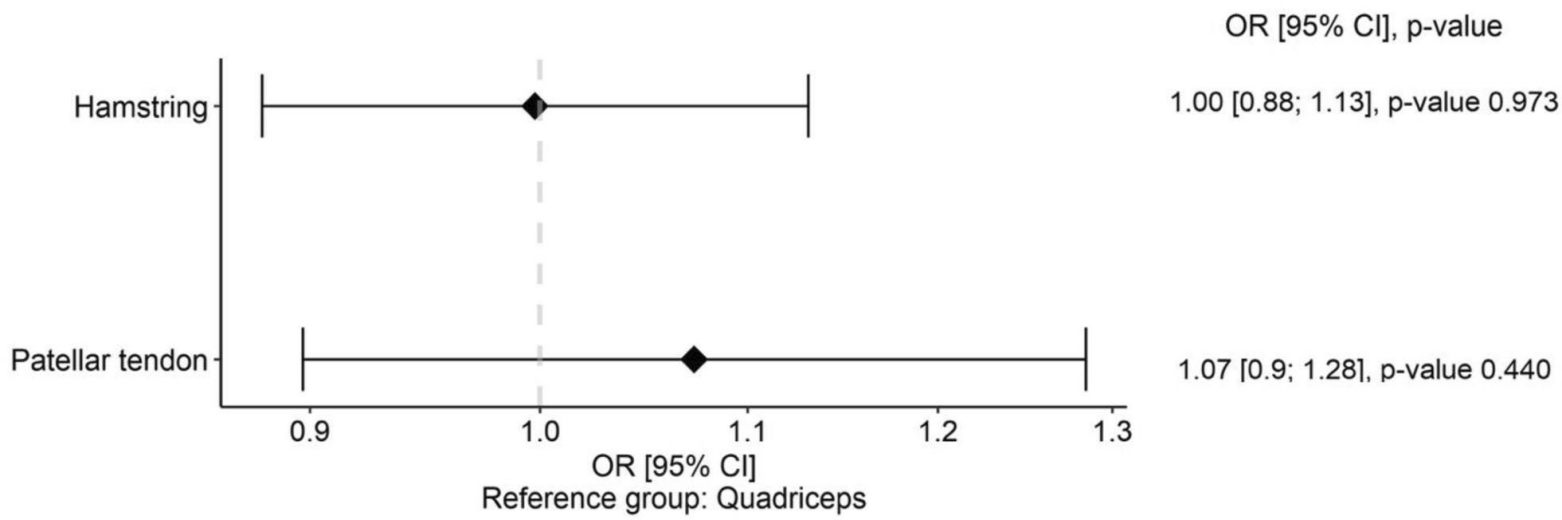
Forest plot of odds ratios (OR) for new knee surgeries

The pooled odds ratio (OR) analysis revealed no significant differences (*P* > 0.05) among graft types for the risk of new knee surgeries, ranging from OR = 1.00 (95% CI 0.88–1.13) for HT to OR = 1.07 (95% CI 0.90–1.28) for BPTB. The corresponding forest plot is presented in Fig. 9.

## Discussion

This meta-analysis of RCTs demonstrates that QT, HT, and BPTB autografts show no statistically significant differences in clinical outcomes after ACLR. Across the 11 RCTs included, mean IKDC and Lysholm scores were consistently high, side-to-side anterior tibial translation was approximately 1 mm, and pooled graft re-rupture and reoperation rates were below 3%. Importantly, no significant differences were found among QT, HT, and BPTB in patient-reported outcome measures (PROMs), objective stability, or complications. These findings corroborate the most recent high-level evidence, including the 2025 systematic review by White et al. [44], which likewise reported no significant differences in graft failure, joint laxity, or complications when comparing the three autografts across randomized trials. Collectively, these results confirm that, in the context of contemporary surgical techniques and rehabilitation, graft selection does not substantially influence mid-term clinical outcomes.

Compared to previous systematic reviews, the present study provides several important contributions to the literature. First, this meta-analysis includes only level-I/II randomized controlled trials, thereby offering the highest-quality evidence currently available and reducing the methodological heterogeneity that has limited prior analyses, many of which combined retrospective cohorts with randomized studies. Second, this work provides a direct, parallel comparison of QT, HT, and BPTB autografts within a single analytical framework. Previous reviews often evaluated QT versus HT or QT versus BPTB separately, limiting the ability to contextualize graft performance across all three commonly used autografts.

Third, we assessed several clinically relevant outcomes using standardized statistical methods, including graft failure, donor-site morbidity, knee stability, and patient-reported outcomes, thereby addressing gaps in earlier studies that frequently reported fragmented or selectively analyzed endpoints. Finally, by incorporating only contemporary RCTs with modern surgical techniques, this study reflects current clinical practice more accurately than prior reviews that included older procedures or heterogeneous rehabilitation protocols. Taken together, these strengths clarify the relative performance of QT, HT, and BPTB grafts and provide an updated, high-level evidence base to inform graft selection in ACL reconstruction.

The present findings are highly consistent with and extend prior meta-analyses. Mouarbes et al. [23] first demonstrated comparable stability and PROMs among QT, HT, and BPTB autografts, though their analysis included mostly level III evidence. Subsequent high-quality reviews, such as those by Dai et al. [22] and Ajrawat et al. [9], reaffirmed the equivalence of QT with the more established HT and BPTB autografts. More recently, Connors et al. [12] reported similar return-to-sport and graft-failure rates between BPTB and HT, while Kurkowski et al. [45] specifically focused on QT and confirmed that, at five or more years of follow-up, QT reconstruction yields clinical outcomes comparable to both BPTB and HT. The convergence of these independent meta-analyses—spanning a range of populations, surgical techniques, and follow-up durations—underscores the robustness of the conclusion that no single autograft offers superior clinical efficacy.

Donor-site morbidity has historically represented a key consideration in graft selection. Although BPTB harvest has traditionally been associated with higher rates of anterior knee pain and kneeling discomfort, as shown in classic studies and recent systematic reviews, our meta-analysis was unable to pool donor-site morbidity data for BPTB because this outcome was not consistently reported across the included RCTs [14, 46]. As a result, direct comparisons involving BPTB could not be quantified in the present analysis. In contrast, HT and QT autografts demonstrated comparable donor-site morbidity based on the available randomized evidence, and recent observational studies further suggest that contemporary minimally invasive QT harvest techniques may reduce anterior knee symptoms and quadriceps weakness. Kunze et al. [14], in a network meta-analysis of RCTs, found significantly lower donor-site morbidity with HT and QT than with BPTB, without compromising graft stability. Recent evidence further clarifies the comparison between HT and QT: Giusti et al. [47] reported comparable donor-site morbidity between all soft tissue QT and HT autografts in a 2025 monocentric observational study, while Runer et al. [20] confirmed that minimally invasive QT harvest techniques achieve excellent outcomes with minimal anterior knee pain and quadriceps weakness. The meta-analysis by Dai et al. [22] directly comparing QT and BPTB also demonstrated lower kneeling discomfort and anterior knee pain with QT. Therefore, while historical evidence indicates that BPTB may carry a higher burden of anterior knee morbidity, this interpretation should be made cautiously, given the lack of analyzable donor-site morbidity data for BPTB within the present study.

Beyond clinical morbidity, intrinsic biomechanical characteristics of grafts have long influenced surgical choice. Structural and ultrastructural analyses provide key insights. Hadjicostas et al. [48] demonstrated that QT and BPTB autografts share a collagen fiber orientation and cross-sectional architecture closer to the native ACL than HT, while Banovetz et al. [49] detailed the anatomic dimensions and insertional footprints of the ACL and common autografts, showing that QT offers the largest mean cross-sectional area. In a systematic review, Malige et al. [50] found that QT and BPTB exhibit higher ultimate load to failure and stiffness compared to HT, which, despite excellent tensile strength, has a more variable diameter.

Current trends in graft utilization help contextualize these findings. Recent registry, survey, and epidemiologic reports indicate a steady global increase in the use of the quadriceps tendon for primary ACL reconstruction [51–54]. Although hamstring and BPTB autografts remain widely employed, several contemporary analyses—including benchmarking studies and surveys of high-volume ACL surgeons—show that QT has transitioned from an emerging graft to a mainstream option. This shift likely reflects growing surgeon confidence supported by accumulating evidence that QT provides clinical outcomes comparable to those of traditional autografts, as demonstrated in the present RCT-based meta-analysis [51–54].

Although biological differences in graft healing kinetics exist—such as the bone-to-bone incorporation of BPTB compared to the soft-tissue integration of HT and QT—experimental evidence suggests that all commonly used autografts ultimately achieve sufficient remodeling and structural strength. These theoretical distinctions did not translate into clinically meaningful differences in the RCTs included in this meta-analysis, consistent with our findings of comparable postoperative stability and patient-reported outcomes across graft types [50, 55, 56].

These findings have several clinical implications. Given the absence of statistically significant differences in clinical outcomes and donor-site morbidity between QT and HT in this RCT-based meta-analysis, graft selection should primarily be individualized according to patient anatomy, surgical history, sport demands, and surgeon experience rather than assumptions about donor-site symptoms [53, 54]. Although prior literature has suggested potential advantages of QT or HT for individuals whose occupations or sports involve frequent kneeling, our pooled analysis does not demonstrate a difference between these two soft-tissue grafts; therefore, such considerations should be interpreted with caution [14, 22]. QT may remain a reasonable option in scenarios such as revision surgery, prior hamstring harvest, or when a larger graft diameter is desired, while BPTB may still be preferred in athletes prioritizing bone-to-bone healing and accepting the risk of anterior knee discomfort. Ultimately, the comparable outcomes across graft types observed in this review support a shared decision-making approach tailored to patient-specific priorities and clinical context.

Several limitations of the current meta-analysis should be acknowledged. Although all included studies were randomized, most were rated as having some concerns for bias, primarily related to incomplete blinding of outcome assessors and inconsistent prospective registration. Loss to follow-up exceeding 20% in at least one trial further introduces the possibility of attrition bias. Heterogeneity in surgical techniques (single-bundle versus double-bundle reconstruction, transtibial versus anteromedial portal drilling, with or without bone-block), fixation methods, and rehabilitation protocols is another potential confounder, although sensitivity analyses failed to detect significant subgroup effects. Moreover, long-term data remain limited; only a minority of included trials reported follow-up beyond 10 years, leaving unanswered questions regarding the incidence of late graft failure, persistent donor-site symptoms, and the development of post-traumatic osteoarthritis [57].

A key limitation is the extremely low overall re-rupture rate (0.7%), which restricts the ability to perform meaningful heterogeneity testing and reduces the statistical power to detect between-group differences. As with any outcome characterized by sparse events, the interpretation of pooled estimates should therefore be made with caution.

Small-study effects should also be considered when interpreting the present findings. Funnel and DOI plots demonstrated notable asymmetry for several outcomes, including IKDC (LFK index −4.49), Lysholm score (LFK index −2.4), and KT-1000 laxity (LFK index −3.98), suggesting the possible presence of small-study effects. Although trim-and-fill sensitivity analyses did not substantially alter the pooled estimates, the observed asymmetry indicates that the available evidence base remains limited and may influence the precision of pooled results. Therefore, the absence of statistically significant differences among graft types should be interpreted cautiously.

Another important limitation is the heterogeneity in follow-up duration among the included randomized controlled trials, which ranged from short-term to long-term assessments. This variability may influence outcomes such as graft failure, reoperation rates, and the detection of late complications. In particular, shorter follow-up periods may underestimate late graft failures or degenerative changes, potentially contributing to the very low pooled event rates observed in the present analysis. Therefore, the absence of statistically significant differences among graft types should be interpreted in the context of this follow-up heterogeneity.

Finally, because the included RCTs do not form a fully connected evidence network and the analysis was not a formal network meta-analysis, our comparisons across QT, HT, and BPTB rely on pairwise data and mixed-effects meta-regression rather than true three-way comparative modeling. Therefore, cross-graft comparisons should be interpreted cautiously and do not imply full equivalence among all three graft types.

Future research should address several persistent gaps. Large, multicenter randomized controlled trials with standardized surgical techniques, rehabilitation protocols, and long-term follow-up are needed to determine whether subtle differences in graft biology influence outcomes such as graft survival or osteoarthritis development. Further investigation into biologic augmentation strategies—including growth factors, stem-cell-based approaches, and scaffold-assisted grafts—may help optimize graft integration and long-term knee health. Emerging data-driven tools, including early applications of artificial intelligence, may eventually support more individualized graft selection, although this remains an exploratory area requiring dedicated study [58, 59].

## Conclusions

This meta-analysis of level-I/II randomized controlled trials found no statistically significant differences among quadriceps tendon, hamstring tendon, and bone–patellar tendon–bone autografts in patient-reported outcomes, knee stability, graft re-rupture, or additional knee surgery after ACL reconstruction. However, these findings should be interpreted cautiously given the limited number of randomized trials and the presence of potential small-study effects.

These results suggest that contemporary surgical techniques and rehabilitation protocols may minimize the clinical impact of intrinsic graft-specific biomechanical differences. Therefore, graft selection should be individualized based on patient characteristics, previous surgeries, sport or occupational demands, and surgeon experience rather than expectations of superior objective outcomes.

Although the current evidence is robust for mid-term follow-up, future studies should include long-term follow-up (≥ 10–15 years), use standardized and validated PROMs, incorporate objective strength assessments, and apply uniform scoring systems for donor-site morbidity. Such methodologically harmonized studies are necessary to clarify the incidence of late graft failure, post-traumatic osteoarthritis, and potential subgroup effects that may not be detectable in shorter follow-up periods.

## Supplementary Information

Supplementary Material 1.

## Data Availability

Raw data are available upon request to the corresponding author.
