# Supplementary material for: Clinical outcomes of quadriceps, hamstring, and bone–patellar tendon–bone autografts for ACL reconstruction: a meta-analysis of randomized controlled trials
Source: Knee Surg Relat Res. 2026 May 7;38:19. doi: 10.1186/s43019-026-00320-w (PMC13154517; doi:10.1186/s43019-026-00320-w)
Supplement: Supplementary file 1 — Supplementary Material 1. [file 43019_2026_320_MOESM1_ESM.docx]

# Meta-analysis: IKDC

Grafici: *Forest1* e *Funnel1 e Doi1*

| **Characteristics of the metaregression** | **Values** |
| --- | --- |
| Tau^2^ | 0.006 (SE = 0.002) |
| R^2^ | 1.05% |
| **Test for subgroup differences** |  |
| Between group | Q_2_=2.7, p-value:0.255 |
| Within group | Q_17_=198.2, p-value<0.001* |

Estimates

|  | N of studies | Mean [95%CI] | Subgroup comparison  (p-value) |
| --- | --- | --- | --- |
| Quadriceps | 10 | 85.67 [81.55; 89.99] | Reference group |
| Hamstring | 7 | 86.07 [81.19; 91.25] | 0.903 |
| Patellar tendon | 3 | 78.89 [71.88; 86.58] | 0.125 |

## 2.4 Funnel plot & doi plot

| Rank correlation test | p-value: <0.001* |
| --- | --- |
| Linear regression test | p-value: <0.001* |
| Doi plot | major asymmetry |
| LFK index | -4.49 |

**By group**

|  | Rank correlation test (p-value) | Egger’s Regression Test (p-value) | Doi plot asymmetry (interpretation) | LFK index |
| --- | --- | --- | --- | --- |
| Quadriceps | 0.073 | 0.124 | Major asymmetry | -3.84 |
| Hamstring | 0.069 | <0.001* | Major asymmetry | -3.63 |
| Patellar tendon | >0.999 | 0.269 | Major asymmetry | -5.34 |

## 2.5 Sensitivity analysis

- Overall estimate with Trim-and-fill method

Number of studies added: 7

Number of studies: 27

|  | Estimate [95%CI] |
| --- | --- |
| Pooled mean | 89.59 [85.53; 93.85] |
| **Heterogeneity** |  |
| Tau^2^ | 0.014 [0.009; 0.029] |
| I^2^ | 94.6% [93.2%; 95.8%] |

**Test of heterogeneity:** p<0.001*

- Fail-safe N Calculation Using the Rosenthal Approach

Fail-safe N: 9882009

# Meta-analysis: Lysholm

| **Characteristics of the model** | **Values** |
| --- | --- |
| Tau^2^ | 0.001 (SE = 0.0004) |
| R^2^ | 0.0% |
| **Test for subgroup differences** |  |
| Between group | Q_2_=0.8, p-value:0.686 |
| Within group | Q_11_=60.7, p-value<0.001* |

Estimates

|  | N of studies | Mean [95%CI] | Subgroup comparison  (p-value) |
| --- | --- | --- | --- |
| Quadriceps | 7 | 92.43 [90.32; 94.60] | Reference group |
| Hamstring | 5 | 93.41 [90.80; 96.11] | 0.572 |
| Patellar tendon | 2 | 94.32 [90.26; 98.55] | 0.426 |

## 3.4 Funnel plot & doi plot

| Rank correlation test | p-value: 0.127 |
| --- | --- |
| Linear regression test | p-value: 0.006* |
| Doi plot | Major asymmetry |
| LFK index | -2.4 |

**By group**

|  | Rank correlation test (p-value) | Egger’s Regression Test (p-value) | Doi plot asymmetry (interpretation) | LFK index |
| --- | --- | --- | --- | --- |
| Quadriceps | >0.99 | 0.663 | Minor asymmetry | -1.1 |
| Hamstring | 0.017* | <0.001* | Major asymmetry | -3.5 |
| Patellar tendon | - | - | Minor asymmetry | -1.9 |

## 3.5 Sensitivity analysis

- Overall estimate with Trim-and-fill method

Number of studies added: 5

Number of studies: 19

|  | Estimate [95%CI] |
| --- | --- |
| Pooled mean | 94.77 [92.83; 96.74] |
| **Heterogeneity** |  |
| tau^2^ | 0.002 [0.001; 0.006] |
| I^2^ | 87.9% [82.6%; 91.6%] |

**Test of heterogeneity:** p<0.001*

- Fail-safe N Calculation Using the Rosenthal Approach

Fail-safe N: 21418054

# Meta-analysis: KT-1000 (mm)

## 4.4 Funnel plot & doi plot

| Rank correlation test | p-value: 0.862 |
| --- | --- |
| Linear regression test | p-value: 0.023* |
| Doi plot | Major asymmetry |
| LFK index | -3.98 |

**By group**

|  | Rank correlation test (p-value) | Egger’s Regression Test (p-value) | Doi plot asymmetry (interpretation) | LFK index |
| --- | --- | --- | --- | --- |
| Quadriceps | 0.483 | >0.99 | Major asymmetry | -5.74 |
| Hamstring | 0.004* | 0.398 | Major asymmetry | -4.11 |
| Patellar tendon | - | - | Major asymmetry | -3.00 |

## 4.5 Sensitivity analysis

- Overall estimate with Trim-and-fill method

Number of studies added: 5

Number of studies: 15

|  | Estimate [95%CI] |
| --- | --- |
| Pooled mean | 1.44 [1.16; 1.78] |
| **Heterogeneity** |  |
| tau^2^ | 0.093 [0.022; 0.540] |
| I^2^ | 67.9% [45.1%; 81.3%] |

**Test of heterogeneity:** p<0.001*

- Fail-safe N Calculation Using the Rosenthal Approach

Fail-safe N: 49

# Meta-analysis: Donor Site Morbidity

| **Characteristics of the model** | **Values** |
| --- | --- |
| Tau^2^ | 0.110 (SE = 0.132) |
| R^2^ | 3.1% |
| **Test for subgroup differences** |  |
| Between group | Q_1_=1.1, p-value:0.306 |
| Within group | Q_2_=14.3, p-value<0.001* |

Estimates

|  | N of studies | Mean [95%CI] | Subgroup comparison  (p-value) |
| --- | --- | --- | --- |
| Quadriceps | 2 | 11.36 [6.76; 19.08] | Reference group |
| Hamstring | 2 | 16.48 [10.11; 26.87] | 0.306 |

## 5.4 Funnel plot & doi plot

| Rank correlation test | p-value: 0.750 |
| --- | --- |
| Linear regression test | p-value: 0.388 |
| Doi plot | no asymmetry |
| LFK index | -0.54 |

## 5.5 Sensitivity analysis

- Overall estimate with Trim-and-fill method

Number of studies added: 1

Number of studies: 5

|  | Estimate [95%CI] |
| --- | --- |
| Pooled mean | 15.86 [10.74; 23.42] |
| **Heterogeneity** |  |
| tau^2^ | 0.175 [0.048; 1.629] |
| I^2^ | 88.5% [75.9%; 94.6%] |

**Test of heterogeneity:** p<0.001*

- Fail-safe N Calculation Using the Rosenthal Approach

Fail-safe N: 2039

# Meta-analysis: % of rerupture

| **Characteristics of the model** | **Values** |
| --- | --- |
| tau^2^ | 0.003 (SE = 0.004) |
| R^2^ | 0.0% |
| **Test for subgroup differences** |  |
| Between group | Q_2_= 0.05, p-value=0.975 |
| Within group | Q_19_=25.5, p-value=0.145 |

**Risultati espressi in percentuali**

## 6.3 Funnel plot & doi plot

| Rank correlation test | p-value: 0.004* |
| --- | --- |
| Linear regression test | p-value: 0.020* |
| Doi plot | major asymmetry |
| LFK index | 2.06 |

**By group**

|  | Rank correlation test (p-value) | Egger’s Regression Test (p-value) | Doi plot asymmetry (interpretation) | LFK index |
| --- | --- | --- | --- | --- |
| Quadriceps | 0.178 | 0.310 | Minor asymmetry | 1.64 |
| Hamstring | 0.103 | 0.157 | Major asymmetry | 2.73 |
| Patellar tendon | 0.117 | 0.400 | Minor asymmetry | 1.88 |

## 6.4 Sensitivity analysis

1. Overall estimate with Trim-and-fill method

Number of studies added: 0

Number of studies: 22

1. Fail-safe N Calculation Using the Rosenthal Approach

Fail-safe N: 479

# Meta-analysis: % of New Knee Surgeries

|  | Estimate [95%CI] |
| --- | --- |
| Pooled percentage (Random effects model) | 2.3 [0.6; 4.7] |
| **Heterogeneity** |  |
| tau^2^ | 0.009 [<0.001; 0.023] |
| I^2^ | 51.2% [20.5%; 70.1%] |

**Test of heterogeneity:** Q_21_= 43.1, p=0.003*

**7.2 Metanalisi by group**

| **Characteristics of the model** | **Values** |
| --- | --- |
| tau^2^ | 0.010 (SE = 0.006) |
| R^2^ | 0.0% |
| **Test for subgroup differences** |  |
| Between group | Q_2_= 0.67, p-value=0.714 |
| Within group | Q_19_=42.59, p-value=0.002 |

## 7.3 Funnel plot & doi plot

| Rank correlation test | p-value: 0.514 |
| --- | --- |
| Linear regression test | p-value: 0.864 |
| Doi plot | major asymmetry |
| LFK index | 2.64 |

**By group**

|  | Rank correlation test (p-value) | Egger’s Regression Test (p-value) | Doi plot asymmetry (interpretation) | LFK index |
| --- | --- | --- | --- | --- |
| Quadriceps | 0.936 | 0.837 | Minor asymmetry | 1.46 |
| Hamstring | 0.533 | 0.682 | Minor asymmetry | 1.6 |
| Patellar tendon | 0.602 | 0.195 | no asymmetry | -0.63 |

## 7.4 Sensitivity analysis

1. Overall estimate with Trim-and-fill method

Number of studies added: 0

Number of studies: 22

1. Fail-safe N Calculation Using the Rosenthal Approach

Fail-safe N: 815
